# Endodontic management of maxillary first molar with protostylid: a rare case report

**DOI:** 10.1186/s12903-023-03315-1

**Published:** 2023-08-29

**Authors:** Yang Zhou, Ye Tao

**Affiliations:** 1 Hospital of Stomatology, Guanghua School of Stomatology, Sun Yat-Sen University, No.56, Lingyuan West Road, Guangzhou, 510055 China; 2 Guangdong Provincial Key Laboratory of Stomatology, Sun Yat-Sen University, Guangzhou, China

**Keywords:** Supernumerary cusp, Protostylid, CBCT, Endodontic management

## Abstract

**Background:**

A protostylid is a relatively rare dental developmental aberration characterized as an extra cusp located on the mesial half of the buccal surface of the molars. A protostylid is rarely to be reported due to its low rate of occurrence. This case report describes a patient referred for endodontic treatment due to the presence of a protostylid on the buccal surface of the maxillary first molar that induced apical periodontitis.

**Case presentation:**

A 53-year-old female reported a 3-month history of pain of chewing with her upper left posterior teeth over 3 months. In the clinical examination, an abraded anomalous cusp-like structure was found on the buccal surface of tooth 26, Cone beam computed tomography (CBCT) revealed a supernumerary cusp with an intact root canal inside, which was fused with the mesiobuccal (MB) root canal in the middle of the root. In addition, extensive periapical radiolucency was observed around tooth 26. The tooth was diagnosed as apical periodontitis, and endodontic treatment was performed. The initial lesion in tooth 26 gradually healed over 1 year of observation.

**Conclusions:**

To our knowledge, this case is the first to describe the endodontic management of a maxillary first molar with a protostylid and advances our understanding of supernumerary cusps. This case provides a reference for the treatment of protostylid.

## Background

The protostylid is a supernumerary cusp located on the mesial half of the buccal surface of the molars [1, 2]. The first description of a protostylid was provided by Dahlberg who defined it as “an elevation or ridge of enamel that rises from the gingival end of the buccal groove and extends mesio-occlusally on the anterior part of the buccal surface of the lower molars.” [3, 4]. De Jonge-Cohen then termed it as “mesiobuccal edge prominencies” [1].A protostylid most commonly appears as a surface irregularity, which is divided into eight categories, presenting in the following form: (a) a smooth buccal surface; (b) a buccal fissure with a pit; (c) a buccal fissure curved distally; (d) a distal furrow from the vestibular furrow; (e) a more pronounced secondary groove; (f) a stronger secondary groove; (g) a secondary groove extending across most of the buccal side of the mesiobuccal cusp; (h) a cusp with a free apex [2, 4]. The incidence of protostylid is low, and Bolk described it as a cusp that is usually found on the vestibular surface of the second and third lower molars, and less frequently on the first lower molars [2]. S. V. S. G. Nirmala et al. reported that protostylid was also found on the left maxillary first primary molar [1]. Furthermore, the incidence of protostylid occurrence is estimated at approximately 2% for Asian populations, especially in permanent mandibular molars [5].

The aetiology of protostylid formation remains unknown, but there is a multifactorial aetiology that includes genetic and environmental factors [6, 7]. Recently, some reports have shown that protostylid originate from the expansion of the inner enamel epithelium and focal hyperplasia of peripheral cells of the mesenchymal dental papilla during the morphological differentiation of tooth development [3]. Meanwhile, Mostowska A et al. found that the PAX and MSX genes are responsible for the development of protostylid [8].

Protostylid is rare to be reported, and the fusion of the mesial buccal cusp with a protostylid associated with root canal treatment has not yet been reported. Thus, endodontic management of a protostylid associated with the maxillary first molar is reported in this case.

## Case presentation

**Fig. 1:**
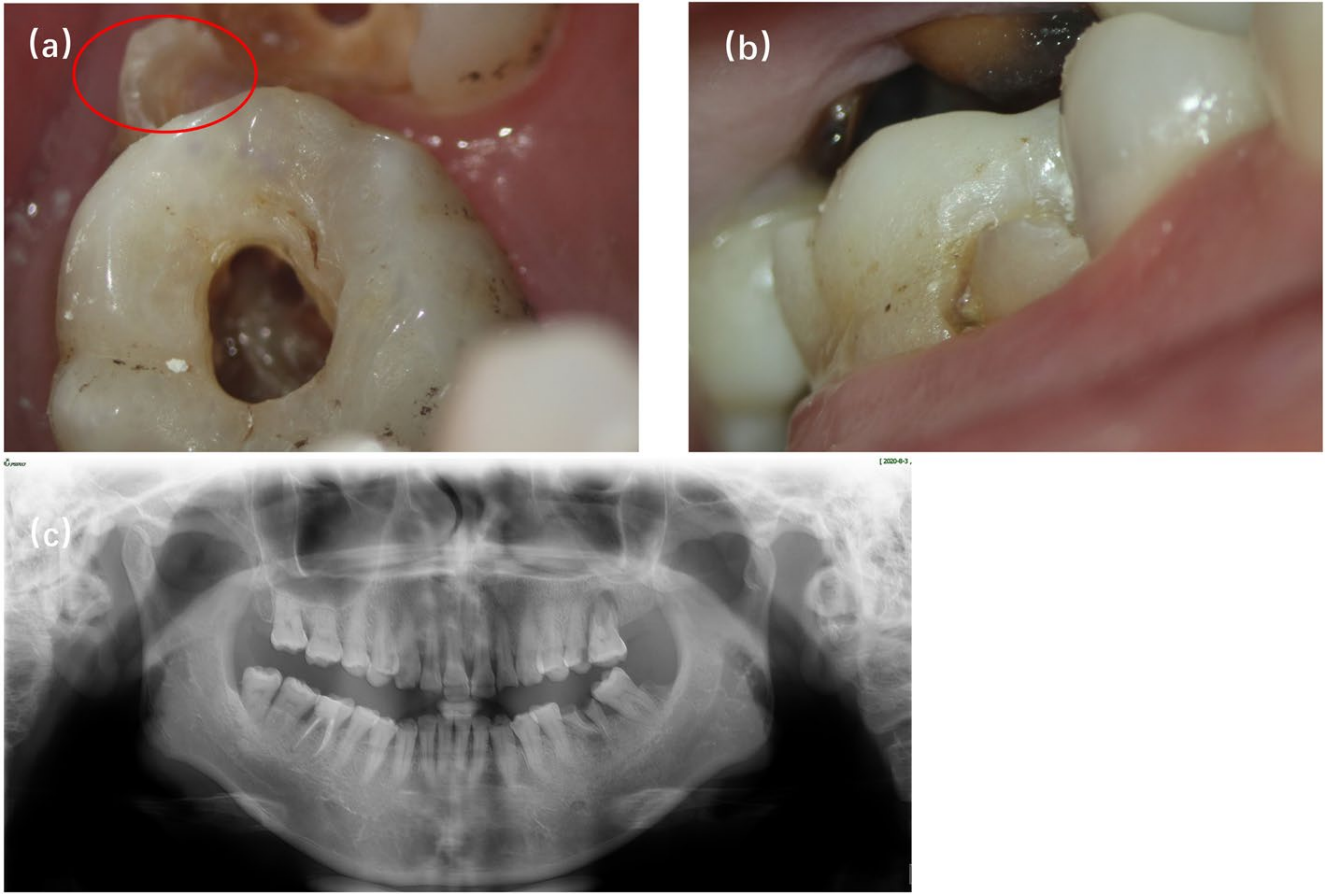
The preoperative photograph and panoramic view. **a**, **b** Preoperative photograph: occlusal and buccal plane of protostylid in tooth 26. Red open circle indicates protostylid. **c** Panoramic view: permanent teeth with normal anatomy

A 53-year-old female patient reported a chief complaint of 3 months of pain upon chewing on her upper left posterior teeth. There was no history of trauma or any hereditary conditions. The medical history of the patient was noncontributory. An abraded anomalous cusp-like structure on the buccal surface of tooth 26 that extended from the cervical edge of the tooth towards the mesiobuccal cusp was discovered during an oral cavity examination (Fig. 1a, b). In addition, four other typical cusps with normal shape and size could be seen from the occlusal view. The tooth showed no response to an electric pulp and cold test, and subsequently, the provisional diagnosis of pulp necrosis was suggested.

**Fig. 2:**
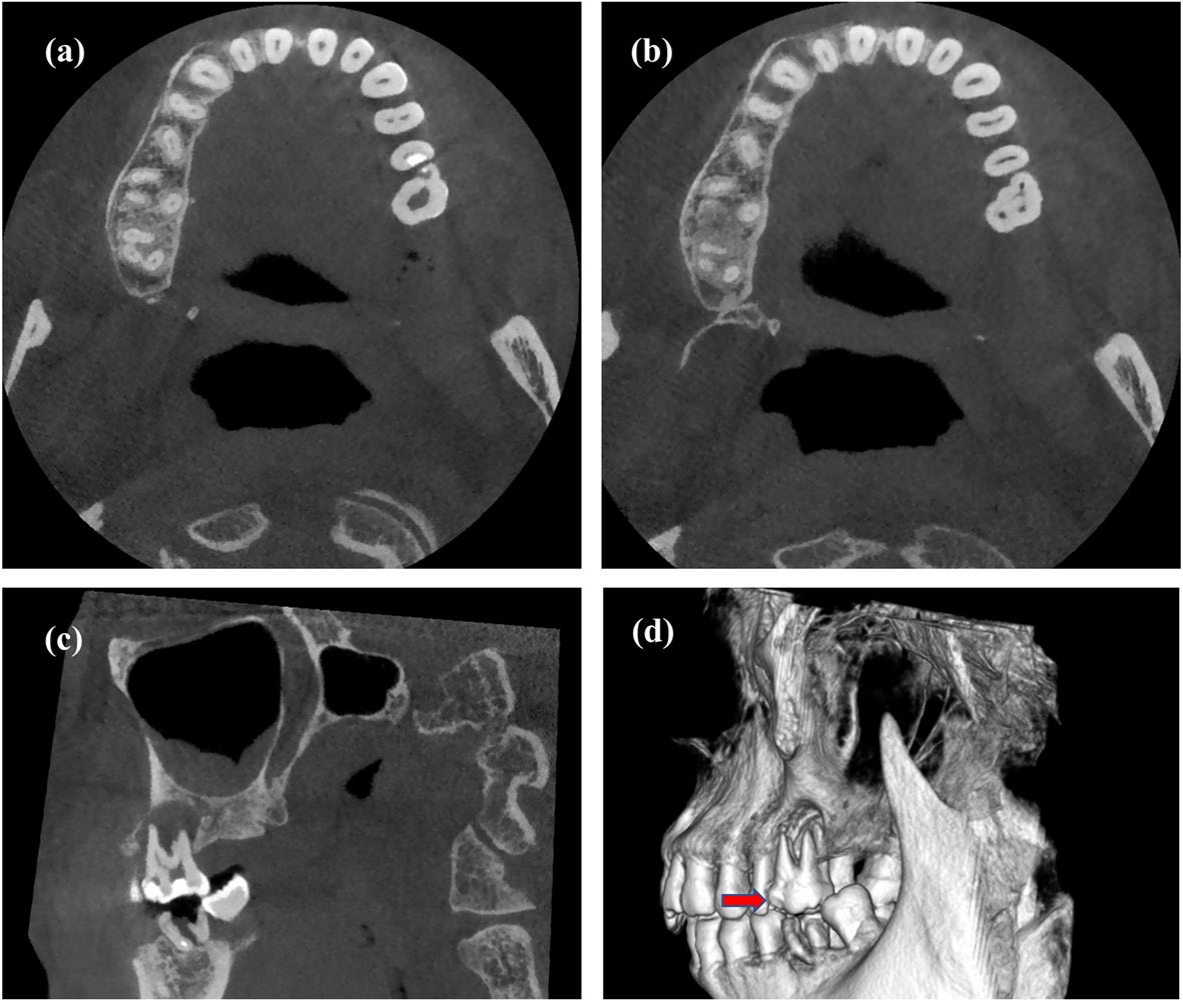
Cone-beam computed tomography images. **a**, **b** Cross section of plane of protostylid in tooth 26; (**c**) Vertebral plane of protostylid in tooth 26; (**d**) 3D reconstruction of protostylid in tooth 26. Red solid arrows indicate protostylid

On radiographic examination, three roots that were generally normal in shape and size were identified, and extensive periapical radiolucency was observed around tooth 26 (Fig. 1c). In addition, extensive periapical radiolucency was observed around tooth 26. However, it was unclear from the radiolucent globe regions if the protostylid had its own root canal. Thus, a cone beam computed tomography (CBCT) (DCTPRO, VATECH, Yongin-Si, Republic of Korea) which was scanned at a voltage of 110 kV and a current of 7.60 mA, was recommended for the patient. The involved tooth was observed, and the CBCT image demonstrated the complex anatomy of tooth 26, in which the pulp chamber of the protostylid was independent in the anterior third of the root (Fig. 2a) and fused with the mesiobuccal (MB) root canal in the apical half of the root (Fig. 2 b, c). A 3D image of tooth 26 was obtained to indicate that the base of projection of the protostylid was related to the mesiobuccal root (Fig. 2d). Therefore, on the basis of the clinical and radiographic findings, the tooth was diagnosed as apical periodontitis according to the AAE’s Guide to Clinical Endodontics [9]. The CBCTPAI score is a scoring system for radiographic assessment of apical periodontitis that has some advantages for clinical applications due to precise measurement of lesion extension and reduced observer interference [10]. In this case, tooth 26 was rated with a score of D due to the extensive destruction of cortical bone. Thus, endodontic treatment of the tooth was planned after the confirmed diagnosis was made.

**Fig. 3:**
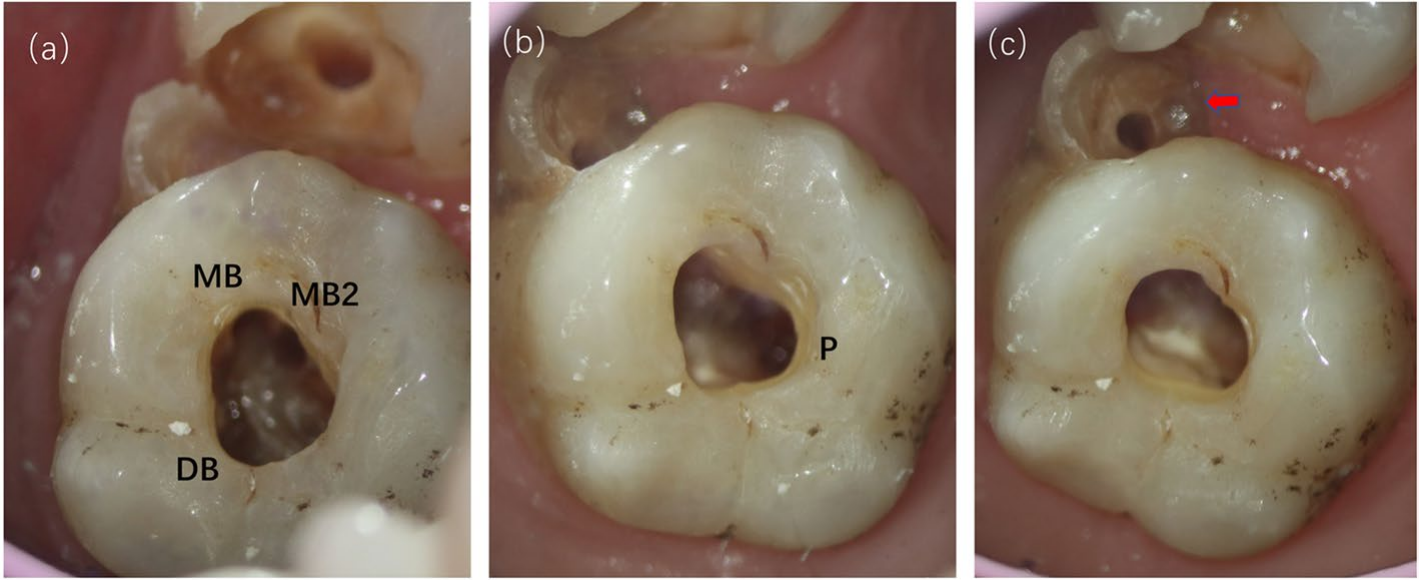
The root canal under operating microscope. Mesiobuccal canal (MB), the second Mesiobuccal canal (MB2), Distalbuccal canal (DB), Palatal canal (P). Red solid arrow indicates the root canal in protostylid

An endodontic access cavity was prepared on the occlusal surface using a fissure bur and a No. 2 round bur. The tooth was isolated immediately upon the discovert of the root canal, following which root canal therapy was conducted. Next, the pulp chamber was examined under 8 × magnification by an operational microscope (OMS2350, ZUMAX, Jiangsu, China). Interestingly, we found that it had 5 root canals: the mesiobuccal (MB) canal, the second mesiobuccal (MB2) canal, the distalbuccal (DB) canal, the palatal (P) canal and the root canal in the protostylid, which is fused with the MB root canal in the middle of the root (Fig. 3). The root canal system was initially prepared with a size 10 C-file (Dentsply Maillefer, Ballaigues, Switzerland) and the working length was determined using a dental apex locator (Raypex6, VDW GmbH, Munich, Germany) combined with a radiograph. Then, the M3 NiTi rotary instruments (M3, United Dental, Shanghai, China) were used to instrument the root canal, and the canals were enlarged by the M3(size 30, 04 taper) to their full working lengths.

During the treatment, the root canal was copiously irrigated with 20 mL of 3% sodium hypochlorite (NaOCl). The root canal was dried with paper points and filled with Vitapex paste (Neo Dental, Tokyo, Japan). Afterwards, the access was sealed with Cavit (3 M ESPE AG, Seefeld, Germany).

**Fig. 4:**
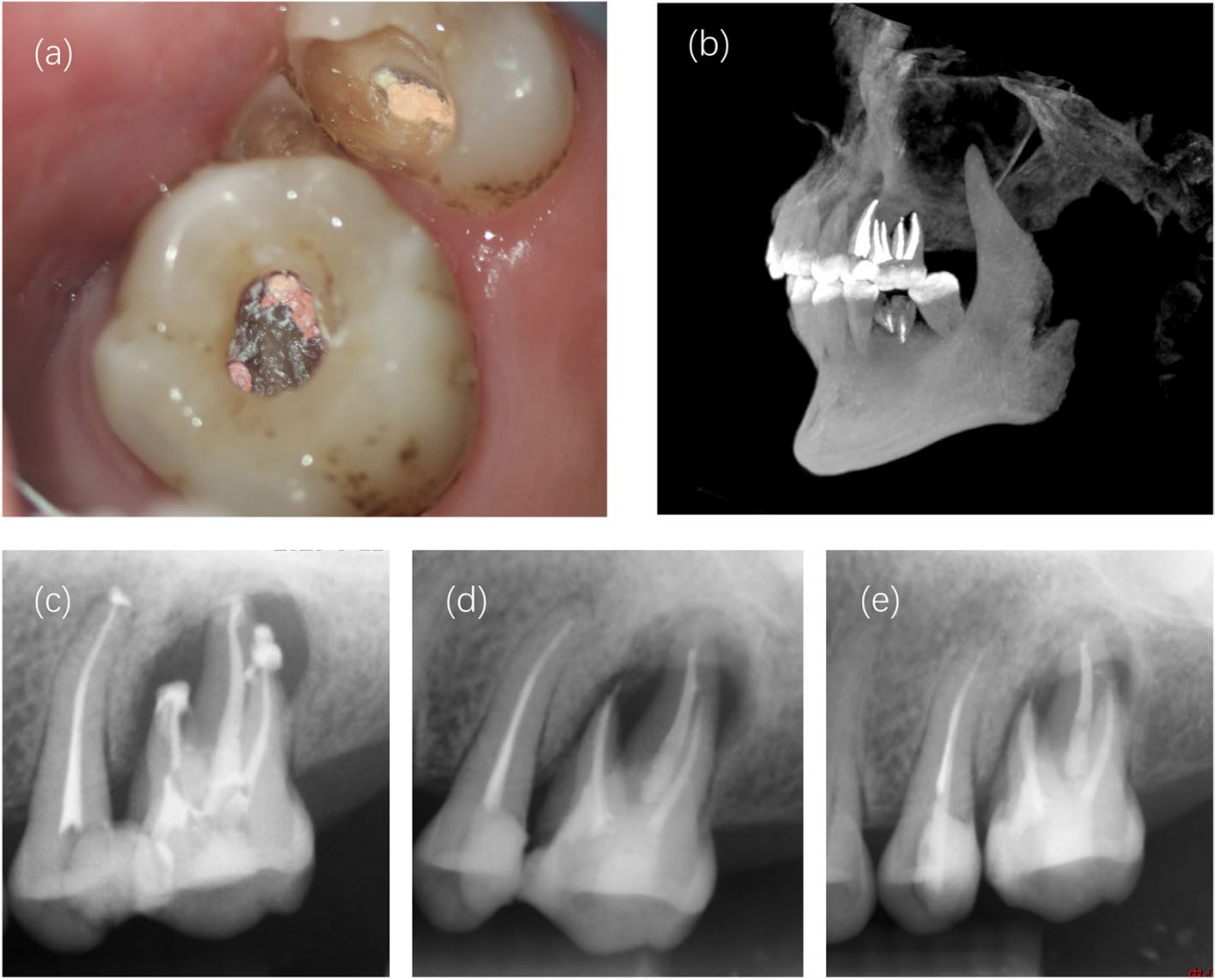
Immediate postoperative and follow-up. **a** The filled root canal under operating microscope; (**b**) 3D reconstruction of filled tooth 26; (**c**) A periapical radiograph taken after treatment; (**d**) 6-month follow-up; (**e**) 1 year follow-up

The patient was called back after 3 months for obturation. Although the clinical examination, it was found that the tooth was functioning and showed no symptoms of oedema. During the appointment, the Vitapex paste was removed using 1% NaOCl, and the root canals were sealed with the iRoot SP root canal sealant (Innovative BioCreamix Inc, Vancouver, Canada) and warm gutta-percha using a vertical condensation technique (Fig. 4a). CBCT (Fig. 4b) and periapical radiographs (Fig. 4c) were taken to test the filling effect. The access cavity was then restored with a posterior composite restoration. (Z350, 3 M Dental Products, St. Paul, Minn).

The patient was scheduled for follow-up visits and seen again at 6 months and 1 year after treatment. At the 6-month follow-up, a periapical radiograph was taken, and the image revealed a reduced apical area (Fig. 4d). At the 1-year follow-up, the tooth remained to be free of symptoms and radiographic evaluation of tooth 26 (Fig. 4e) showed a healing apical area.

## Discussion and conclusions

Human teeth may exhibit various changes in morphological features. Developmental factors determine how teeth develop, particularly the form of new cusps. Thus, initiation and placement of presumptive cusp tips, epithelial folding and mesenchymal growth, termination of crown formation, and initiation of root formation are all factors that determine the shape of the tooth crown [11].

A supernumerary cusp is an abnormality of the tooth shape. A supernumerary cusp usually refers to an accessory cusp on the buccal or lingual surface of a normal crown such as a talon cusp, central cusp or Carabelli cusp [6, 12]. In the clinic, the Carabelli cusp is frequently observed on the palatal surface of the mesiopalatal cusp of the maxillary permanent molars and maxillary second deciduous molars [13]. Similar to the Carabelli cusp, a rare supernumerary cusp called a protostylid usually appears as a pit and distal bending of the buccal groove or surface irregularity [5]. In the present case, the protostylid was reported to be located on the mesial half of the buccal surface on the first molar.

The aetiology of protostylid remains unknown, but various theories have been proposed to explain their existence. Some researchers have hypothesized that protostylid originate from the growing centre of an enamel knot that will eventually become the location of the cuspid vertices during the morphogenetic phase, which is before the onset of amelogenesis and dentinogenesis [2, 14].

It should be noted that the diagnosis of a protostylid should be determined carefully because it is difficult to be differentiate between a fused tooth and a concrescent tooth. Generally, fusion results from the union of two or more developing contiguous dental germs at the enamel, dentin, and cementum, which is usually observed in primary and permanent dentition and particularly appears in the anterior position [15, 16]. A concrescent tooth is an uncommon abnormality with two crowns and double roots that arises in the maxillary second or third molar [17]. Thus, accessory oral examinations such as CBCT are extremely useful for antidiastole. CBCT is a contemporary computed tomography technology that reconstructs two-dimensional images into three-dimensional images using digital projection of cone beam radial images [18]. Traditional X-ray radiographs make endodontic diagnosis and treatment planning difficult due to their limitations, including anatomic 3D compression, geometric alteration and anatomic obstacles [19]. Hence, CBCT has proven to be quite useful in the localization and identification of the tooth anatomy due to its advantages, such as quick imaging duration, minimal radiation exposure and excellent spatial resolution [18, 20]. Furthermore, CBCT also has known limitations, including the possibility of artefact production, high degrees of scatter and noise, and variability in dosage distribution within a volume of interest [21]. Therefore, the combination of CBCT and traditional X-ray radiographs should be taken into consideration [22]. In this case, CBCT evaluation revealed that the root canal inside the supernumerary cusp had fused with the mesiobuccal root canal in the middle of the root, and extensive periapical radiolucency was observed around tooth 26. This complex variation in root canal morphology and complicated chronic periodontitis poses a great challenge for root canal therapy.

The management of supernumerary cusps is diverse and mainly determined on an individual basis, which includes no treatment, sequential grinding, pit and fissure sealants, pulp therapy, restorative treatment, full crown coverage, and extraction of the affected tooth [23]. Protostylid may cause some common dental pulp diseases, such as pit and fissure caries, sensitivity, and tooth devitalization due to fracture or attrition of the accessory cusp leading to pulpal exposure [14]. Thus, the treatment of protostylid relies on whether the cusp is close to the pulp or contains pulp and requires a comprehensive clinical assessment. In the present case, we found that buccal protostylid were abraded in tooth 26, thus leading to chronic apical periodontitis. In particular, root canal therapy of molars with protostylid should be taken into consideration because their morphological diversity and limited visibility make access and canal orifice identification challenging [24]. The operating microscope has recently been adopted to see the tiniest details inside a patient's tooth, which can magnify images up to 25 times larger than those seen with the unaided eye and is useful in detecting the location of hidden and accessory canals [25–27]. Thus, the endodontic treatment in this case was completed with an operating microscope. Considering that irregular root canal morphology was the deciding factor during the shaping procedures, a large amount of chemical irrigation along with mechanical debridement was performed. Furthermore, root canal disinfection also plays an important role in root canal therapy. Vitapex is a calcium hydroxide paste which composed of calcium hydroxide, iodoform and polysiloxane oil [28, 29]. In the clinical, Vitapex paste is usually applied for root canal disinfection due to the antibacterial properties of calcium hydroxide and iodoform [28]. In this case, due to the extensive apical periodontitis, Vitapex paste was preferred as the intracanal medicament to remove the necrotic residual pulp tissue and eliminate osteoclastic activity. Furthermore, we used the root iRoot SP root canal sealer, which was been shown to have a good biocompatibility, hydrophilicity and slight setting expansion, resulting in decreased apical microleakage [30]. Eyal Rosen et al. showed that most cases of impaired feeling following root canal filling material extrusion appear to fully or partially recover over time [31]. In the present case, a small root canal filling material extrusion appeared around the periapical tissues and was found in the periapical radiograph. After one year of observation, the patient was clinically asymptomatic, and the periapical lesion size was reduced. However, the long-term prognosis regarding extrusion of the material remains to be observed. In general, the absence of pain and other symptoms, the absence of a sinus tract, no loss of function, and radiographic evidence of a normal periodontal ligament space around the root are favourable outcomes of root canal treatment [32]. Root canal treatment should be evaluated at least after one year. If the lesion shown in radiographs has kept the same size or has simply shrunk in size, it is recommended that the lesion should be evaluated further until it has healed or for a minimum period of 4 years [32]. The patient was clinically asymptomatic, and by CBCT evaluation, the extensive destruction of cortical bone around tooth 26, which was rated with a score of D, dropped to a score of 4 (diameter of the periapical radiolucency: > 4–8 mm) after one year, which indicated that the initial lesion in tooth 26 had begun to heal. However, the subsequent recovery situation remains to be observed.

In conclusion, the occurrence of protostylid is a relatively rare phenomenon. To our knowledge, this case is the first to describe the endodontic management of a maxillary first molar with a protostylid. CBCT has significant advantages in the clinical diagnosis. Owing to the aberrant morphology of the crown and the complexity of the root canal system, the treatment procedures for protostylid require special consideration.

## Data Availability

All data generated or analyzed during this case are included in this published article.

## Abbreviations

CBCT: Cone beam computed tomography
MB: Mesiobuccal
MB2: The second mesiobuccal
DB: Distalbuccal
P: Palatal
NaOCl: Sodium hypochlorite
